# Assessment of Functional Performance, Self-Healing Properties and Degradation Resistance of Poly-Lactic Acid and Polyhydroxyalkanoates Composites

**DOI:** 10.3390/polym14050926

**Published:** 2022-02-25

**Authors:** Emanuele Rossi, Arjun Raghavan, Oguzhan Copuroglu, Henk M. Jonkers

**Affiliations:** Department of Materials & Environment, Faculty of Civil Engineering & Geosciences, Delft University of Technology, Stevinweg 1, 2628 CN Delft, The Netherlands; a.raghavan-1@student.tudelft.nl (A.R.); o.copuroglu@tudelft.nl (O.C.); h.m.jonkers@tudelft.nl (H.M.J.)

**Keywords:** polyhydroxyalkanoate, self-healing concrete, waste-derived materials

## Abstract

In this study, the applicability of two bacteria-based healing agents (e.g., poly-lactic acid and polyhydroxyalkanoate) in blast furnace slag cement (BFSC) mortar has been assessed. An experimental campaign on the functional properties, self-healing capacity, freezing–thawing and carbonation resistance has been conducted in comparison with plain mortar (Ctrl). Due to the relatively low alkalinity of the mixture, the addition of poly-lactic acid healing agents (PLA) caused coarsening of the micro-structure, decrease of strength and did not improve the self-healing capacity of the material. Among other consequences, the mass loss due to the freezing–thawing of PLA specimens was about 5% higher than that of the Ctrl specimens. On the contrary, no detrimental effect of the mortar functional properties was measured when polyhydroxyalkanoate healing agents (AKD) were added. The self-healing capacity of AKD specimens was higher than that of the Ctrl specimens, reaching a maximum healed crack width of 559 µm after 168 days of self-healing, while it was 439 µm for the Ctrl specimens and 385 µm for PLA specimens. The air void content of the AKD mixture was 0.9% higher than that of the Ctrl, increasing its resistance against freezing–thawing cycles. This study aims to confirm the potential applicability of AKD particles as self-healing agents in low-alkaline cementitious mixtures.

## 1. Introduction

The durability of concrete is a major issue worldwide. Among many degradation mechanisms that can affect infrastructures, steel reinforcement due to chloride penetration is one of the most detrimental. The risk of reinforced concrete degradation is even more threatening when infrastructures are exposed to coastal or freezing environments due to the higher availability of chlorides in the surrounding seawater or transported through de-icing salts, respectively. To enhance the durability of structures, blast-furnace slag (BFS) cementitious materials showed a good resistance to aggressive environments thanks to its finer capillary porosity and lower chloride migration/diffusion coefficient than that of Ordinary Portland cement (OPC) mixtures [1,2,3]. Furthermore, they are characterized by higher long-term compression strength as well as improved workability [4,5]. Besides these useful durability and functional properties, the use of BFSC is environmentally advantageous, since it halves the CO_2_ footprint of concrete. According to Lauser and Burger, by limiting the clinker content in the mixture, the environmental impact of cement drops from 930 kg of CO_2_ per ton for Ordinary Portland cement to 400 kg of CO_2_ per ton for high-content blast-furnace slag cement (i.e., CEM III/B with slag > 70%) [6]. Given these beneficial properties, it is not surprising that in the Netherlands, which has more than 450 km of coastline, the market share of BFSC is over 60%.

Despite the good performance of BFS concrete in aggressive environments, cracking is an inevitable phenomenon, as it is for OPC concrete, because of the brittle nature and the low tensile strength of both mixtures. Compared to OPC, slag-containing concrete is also more sensitive to cracking due to early-age shrinkage [7,8], which can induce cracks up to 500 µm wide for massive concrete structures [9]. Cracks allow harmful agents (i.e., CO_2_ and chlorides) to penetrate the material, reducing its service life and structural reliability and increasing its repair and maintenance costs. However, micro-cracks generally do not negatively influence the service life of concrete, since it has the capacity to autogenously self-heal cracks up to 100–150 µm [10]. This self-healing capacity (SHC) is dependent on many parameters, such as the type of cement and w/c of the mixture, age of cracking and the crack width. Autogenous healing occurs through the continuous hydration of cement particles and through the precipitation of calcium carbonate resulting from carbonation of the matrix [11]. By self-healing a crack completely, fewer or no harmful agents can penetrate the material and, therefore, the service life of the concrete increases. This statement is confirmed in the case of OPC mixtures by many studies that investigated the beneficial influence that the self-healing of micro-cracks (from 5–10 µm to 60 µm wide cracks) has on the chloride penetration resistance of cracked concrete [12,13,14]. Extending the self-healing capacity of concrete (i.e., self-healing wider cracks as well as self-healing cracks with a given width in a shorter time than how traditional concrete does autogenously) might have a tremendously positive influence on the service life of structures and, therefore, on their repair and maintenance costs. As a consequence, a significant amount of research has been conducted during the last 20 years to improve the self-healing capacity of concrete by means of adding different types of self-healing agents (or self-healing precursors). Among others, the most common healing agents that have been proposed are superabsorbent polymers (SAPs), dispersed organic polymers (i.e., epoxy resin), macro- and micro-encapsulated polymers or minerals (i.e., microfluidics or glass capsules) and, last but not least, bacteria-based precursors that can be dispersed in the matrix, encapsulated (i.e., in hydrogels or polymeric substrates) or impregnated in carriers (i.e., porous granules) [10]. Wang and others [15,16,17] did extensive work on assessing the SHC of mortar with different encapsulated healing agents. In one of their studies [15], they evaluated the effect of a HA composed of melamine-based microcapsules and spores (Bacillus sphaericus LMG 22557) in Portland cement (CEM I 52.5 N) mortar. It is remarkable that the SHC of their system had a maximum healed crack width of 850–970 µm for specimens exposed to water submersion and of 560–600 µm for specimens exposed to wet/dry water cycles. Under the same conditions, their control plain mortar specimens had a maximum healed crack width of 240–250 µm and 75 µm, respectively. Furthermore, the SHC of Portland cement (CEM I 52.5 N) mortar with added-in bacteria and bio-reagents (i.e., yeast extract, urea and calcium nitrate) encapsulated in hydrogels was widely investigated [16,17]. In Wang et al. [16], the SHC of their control plain mortar mixture was 15 ± 10%, while that of the self-healing mortar was around 68%, with a maximum healed crack width of 500 µm after 28 days of incubation (i.e., wet/dry water cycles). In Wang et al. [17], the application of a chitosan-based hydrogel as a self-healing precursor in mortar was investigated and had a SHC equal to 87 ± 5%, while their control plain mortar specimens had 59 ± 10% after 70 days of incubation (i.e., water submersion). Other specimens were exposed to 70 days of wet/dry water cycles, and they could self-heal cracks up to 255 µm wide, while the maximum width that the control specimens could self-heal was 140 µm. Xu et al. [18] investigated the applicability of rubber particles as a bacteria (Sporosarcina pasteurii ATCC11859) carrier in self-healing Portland cement (CEM I PO 42.5) concrete, including the influence that two HA particle sizes have on the properties of the proposed system. The particles ranged between 0.2–0.4 mm (named as series S) and of 1–3 mm (named as series L). The maximum healed crack width for the L-series specimens was 860 µm, that of the S-series was 520 µm and that of the control plain mortar specimens was 300 µm after 28 days of incubation (i.e., water submersion). The regain of compressive strength was also investigated: as a result, the regain of the L-series was 96%, that of the S-series was 90% and that of the control series was 85%.

On the other hand, significantly fewer studies have been conducted on assessing the self-healing capacity of BFSC mixtures. Darquennes et al. [9] investigated the self-healing capacity of BFS mortar specimens in comparison with that of OPC mortar specimens at a different age. The capacity to limit chloride penetration through self-healing of these mixtures was also evaluated. As a result, they reported that the higher the content of slag, the more important the self-healing capacity of the cracked specimens was for crack widths up to 160 µm. The self-healing capacity was also reported to be higher for specimens cracked at early age (i.e., 7 days), since they have a higher content of un-hydrated particles. Moreover, their results show that self-healed specimens could be regarded as specimens characterized by a smaller crack in relation to their chloride penetration resistance. To improve the self-healing capacity of BFS mixtures and, consequently, to seek a more durable engineered material, Palin et al. [19,20,21] incorporated a bacterial isolate and organic mineral precursor compound, as part of a cost-effective agent, for realizing self-healing concrete in low-temperature marine environments. The bacteria-based self-healing cementitious composite displayed an excellent crack healing capacity, reducing the permeability of cracks 400 µm wide by 95% and cracks 600 µm wide by 93%, following 56 days’ submersion in artificial seawater at 8 °C. The self-healing capacity was improved thanks to mineral precipitation as a result of chemical interactions between the cement paste and seawater, bead swelling, magnesium-based precipitates as a result of chemical interactions between the magnesium of the beads and the hydroxide ions of the cement paste and bacteria-induced mineral precipitation. However, even though improving the self-healing capacity of BFS cementitious materials could have significant economic potential, the work of Palin remains isolated overall, since most of the bacteria-based encapsulated techniques proposed in the literature have been assessed with regard to their compatibility and performance in OPC mixtures [15,16,17,18]. Interestingly, Mors and Jonkers [22,23] applied a calcium lactate-based healing agent (PLA) to improve the self-healing capacity of cementitious materials. As a result, it was demonstrated that OPC mortar with added-in 4% w/w cement calcium lactate healing agents could self-heal cracks up to around 650 µm wide and contribute significantly to the surface densification of the matrix. However, when cement was used containing a high slag content as a clinker replacement (CEM III/B, slag > 75%), no surface densification occurred and, also, the material demonstrated increased water absorption. Furthermore, PLA addition to CEM III/B-based material showed significant reduction of the slag reaction and concrete strength development, suggesting the inapplicability of that healing agent to improve the properties of BFS cementitious materials. This incompatibility was the result of the lower pH of BFS pore solution compared to that of OPC mixture, that caused dissolution of the lactates and consequent acidification of the matrix. Parallel to the application of calcium lactate as a self-healing precursor, the applicability in OPC mixtures of an alkanoates-derivates (AKD)-based healing agent derived from waste streams has been previously demonstrated [24,25,26]. Besides improving the self-healing capacity of the mixture [24], its transport properties [26] as well as not negatively influencing the material functional properties [25], AKD has the theoretical potential to also be applied in BFS mixtures, since alkanoates do not need a very alkaline environment (e.g., pH of ~13, like that of OPC) to be metabolized by bacteria and, consequently, to induce self-healing calcium carbonate precipitation. The proposal of healing agents that are compatible with the BFS matrix and that improve its self-healing capacity might open up the possibility to develop a technology with a high potential market value in terms of both service life improvements. On the other hand, the production of CO_2_ due to the bacterial metabolic activity might negatively influence the BFS mixture, coarsening the matrix and decreasing the resistance to, among other mechanisms, freezing and thawing. Hence, this study presents an extensive experimental investigation on the effect of AKD on the functional properties, self-healing capacity and degradation resistance of BFS mixtures. Experiments on the effect that the inclusion of polyhydroxyalkanoate-derived content has on the fundamental properties of mortar have been conducted, namely, its hydration, air void content, setting time, workability, compression and flexural strength. The self-healing capacity of BFS mortar specimens with bacteria-based inclusions has been also assessed through microscopic analysis of the crack closure over time. Finally, the carbonation and freezing–thawing resistance of the mixtures have been evaluated. To seek for a more extensive comparison, the performance of plain BFS mortar as well as of BFS mortar with added-in poly-lactic acid healing agents (PLA) has been also evaluated.

## 2. Materials and Methods

### 2.1. Healing Agents

Alkanoates-derived healing agent (AKD) particles were composed of alkanoate, bacterial spores of Bacillus cohnii-related strains and growth-required nutrients (i.e., yeast extract). Alkanoate-rich biomass was obtained from a pilot plant that uses the organic fraction of municipal waste (OFMSW) as raw material (Orgaworld/Paques, Lelystad, The Netherlands). Under laboratory conditions, the alkanoate was extracted from the biomass using a solvent-based method. Healing agent (HA) particles were obtained by merging together alkanoate, bacterial spores and growth-required nutrients (i.e., yeast extract) through compaction and hot rolling. Prior to testing, the HA particles were ground and sieved, resulting in a particle size ranging between 0.5 mm and 1 mm. More information about the formulation of AKD particles can be found in previously published works [24,25]. A PLA-based healing agent obtained from Basilisk B.V. (Delft, The Netherlands) was composed of lactate derivatives, bacterial spores of Bacillus cohnii-related strains and nutrients. The PLA healing agent was applied in the cementitious system as received.

### 2.2. Assessment of Mortar Functional Properties

The effect of the healing agents on the hydration of blast furnace slag cement (BFSC, CEM III/B 42.5 N, ENCI, Rotterdam, The Netherlands) was investigated through isothermal calorimetry testing. The heat released during cement hydration was monitored for around two days through an eight-channel isothermal calorimeter (TAM Air 3114/2326, thermometric AB, Sollentuna, Sweden), operating at 600 mW and at 20 ± 0.02 °C. In each calorimeter glass ampoule, 2.5 g of cement was mixed with water to obtain a water-to-cement ratio (w/c) of 0.5. PLA and AKD particles were separately added to the paste at 2.6% by mass of cement. One reference sample (Ctrl) for each cement type was also analyzed without any HA addition. For each sample composition, two replicates were tested. The first hour of heat flow measurements were subtracted from the heat curve since they were related to the stabilization of the sample inside the calorimeter. The composition of each tested mixture is reported in Table 1.

To investigate the effect that healing agents have on mortar compression and flexural strength, three series of specimens were cast with blast furnace slag cement (BFSC, CEM III/B 42.5 N, ENCI, Rotterdam, The Netherlands), a water/cement ratio of 0.5 and fine siliceous aggregates (0.125–2 mm). The three series were labelled as follows: Ctrl for plain mortar, PLA for OPC mortar with 2.6% by mass of cement of PLA-based healing agents and AKD for OPC mortar with 2.6% by mass of cement of alkanoates-derived healing agent. Mortar strength was measured according to the procedure described in EN 196-1 [27]. Prismatic specimens of 40 × 40 × 160 mm^3^ were cast and sealed with plastic foil for 24 h before being de-molded. Three-points bending and uniaxial compression tests were conducted at 1, 3, 7 and 28 days of curing, which occurred in a high humidity chamber (RH > 95%). The mixing proportions of the mortar specimens are reported in Table 2 and Table 3. For each mixture, the workability, air void content and (initial and final) setting time were tested according to ASTM C143 [28], ASTM C185 [29] and the Vicat test reported in EN 196-3 [30], respectively.

The pore size distribution and pore volume of specimens derived from each mixture was measured through mercury intrusion porosimetry (MIP). After casting, de-molding and curing for 28 days (T = 20 °C and RH > 95%) as previously described, the central portion of one additional 40 × 40 × 160 mm^3^ prismatic specimen of each mixture was manually fragmented with a chisel and left drying in a vacuum freezer for pore water removal until no further mass loss was measurable (i.e., around 10 days). Then, the pore size distribution and pore volume of around 8 g of fragments of each mixture were determined through MIP using a Micromeritics AutoPore IV mercury porosimeter.

### 2.3. Assessment of Self-Healing Capacity through Microscopy Analysis

The effect of PLA and AKD healing agents on the self-healing capacity of CEM III/B mortar, also depending on the age of cracking, was also investigated through light microscopy analysis. For this test, three mortars were cast, labelled as Ctrl for plain BFS mortar, PLA for BFS mortar with 2.6% by mass of cement of PLA-based healing agents and AKD for BFS mortar with 2.6% by mass of cement of alkanoates-derived healing agent. For each mixture, cylindrical specimens of 35 mm in diameter and 60 mm high were cast with two lateral notches to eventually facilitate the mechanical induction of cracks. After 24 h curing, specimens were carefully de-molded, tightly sealed in plastic foil to avoid the evaporation of water, and kept at room temperature for further curing. For each mixture, the specimens were grouped depending on their age at which they were manually cracked. The first group of specimens was cracked at 7 days of age (labelled as “Mixture-7d”, where mixture indicates Ctrl, PLA or AKD), while the second group of specimens was cracked at 28 days of age (labelled as “Mixture-28d”). Specimen cracking was conducted through a splitting test. After cracking, two plastic spacers were placed at the notches of each specimen to keep the two sides distant from each other. To have cracks with different widths among the specimens, the spacers had a thickness ranging from 0.1 mm to 0.8 mm. After placing the spacers, the sides of the specimens were carefully sealed with tape and silicone. Just after cracking, optical microscope (Leica MZ6, Nussloch, Germany) images of the upper side of each specimen were collected. The initial crack width was calculated as the average of ten crack width measurements taken at around 200 µm of distance between each other. To measure the self-healing capacity of each specimen, the same procedure was conducted after 28, 56, 112 and 168 days of self-healing incubation (storing the specimens in a high humidity room at 20 °C and RH > 95%). As a result, the self-healing capacity (SHC_LM_) was calculated according to Equation (1):

SHC_LM_ (%) = [(w_i_ − w_t_)/w_i_]×100 (1)

where w_i_ is the initial effective crack width measured just after cracking (µm), w_t_ is the crack width measured over the self-healing period (µm) and t is the self-healing duration at which measurements were collected (28, 56, 112 and 168 days).

Further environmental scanning electron microscope (ESEM) analysis was conducted to observe self-healing along the crack depth for one specimen of each mixture (for each age of cracking). The specimens were impregnated with fluorescent epoxy resin and sawn perpendicularly to the crack as soon as the self-healing period was terminated. After sawing, the surface of the sawn samples was again impregnated with epoxy resin. The surface of the specimens was manually ground with #120, #220, #320, #600, #800 and #1200 grinding silicon carbide sanding paper and polished with 6, 3, 1 and 0.25 um polishing diamond paste on a lapping table to obtain a mirror-like surface on the specimens. Each grinding and polishing step took around 10 min. Ethanol and non-water-based polishing paste were used for grinding and polishing, respectively, to avoid further ongoing hydration of the sample. ESEM analysis (ESEM, FEI, Quanta FEG 650) under back-scatter electron mode (BSE) of the polished section obtained perpendicularly to the crack was then conducted. Micrograph acquisition was conducted at 100× magnification and 15.0 kV.

### 2.4. Freezing–Thawing Resistance of Carbonated Specimens

The resistance to the dual attack of carbonation and salt frost attack of the Ctrl, PLA and AKD blast furnace slag mortar specimens was also investigated. Six prismatic specimens of 40 × 40 × 160 mm^3^ were cast for each mortar mixture, sealed with plastic foil for 24 h before being de-molded and later stored in a high humidity chamber (RH > 95%) for 28 days. The specimens had the same composition reported in Table 2 and Table 3. At 28 days of age, the specimens were left non-sheltered and exposed to natural atmospheric carbonation for roughly 11 months (mean monthly RH of 84% and 0.03–0.04% CO_2_). After 11 months’ exposure to natural carbonation, three specimens of each mixture were split in two equal halves (40 × 40 × 80 mm^3^). The freshly split surface of one half was used to demarcate the carbonation front by spraying 1% phenolphthalein solution, wherein the carbonated area was colorless, and the non-carbonated area was rendered shades of pink to purple. The other half was used to pulverize the mortar along the perimeter of the specimen according to the carbonation depth reading of the first half. The material was ground by hand in an agate mortar–pestle, and methanol was added to prevent agglomeration and promote the fineness of the particle sizes to around 10–30 µm before conducting X-ray diffraction analysis (XRD, Philips PW 1830-XRD, Eindhoven, The Netherlands) to identify the carbonated phases. The powdered samples were placed in the aluminum holder by front loading and were subjected to a CuKa radiation with tube settings at 45 kV and 40 mA. The samples were scanned in the range of 5–70° with a step size of 0.030° and at a rate of 3 s per step for data collection.

**Figure 1:**
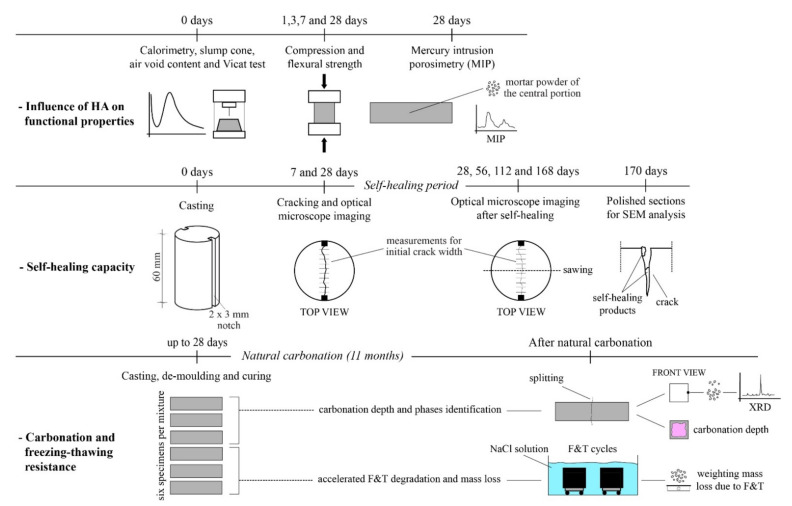
Schematic representation of the whole experimental procedure.

The mass loss due to the (accelerated) freezing–thawing of the other three naturally carbonated specimens was also measured. After 11 months of natural carbonation, the specimens were sawn into cubes of approximately 40 × 40 × 40 mm^3^. Later, the cubes were tightly sealed with plastic tape at the sides, leaving the 3 mm high upper portion of the specimen exposed. To avoid any effect of surface inhomogeneity on the test results [31], the sawn surface was the one left exposed to the freezing medium. The specimen was submerged in 3% NaCl solution to simulate de-icing salt frost attack. To obtain significant damage due to freezing–thawing, the specimens were exposed to cycles of 20 h freezing at −20 °C and 4 h thawing at 25 °C. A total of 28 freeze–thaw cycles were conducted. The scaled mass of each specimen was collected, dried and weighed at the end of the 1st, 3rd, 7th, 10th, 14th, 17th, 21st, 24th and 28th cycle. A schematic representation of the whole experimental campaign is visible in Figure 1.

## 3. Results

### 3.1. Effect of Healing Agents on Mortar Functional Properties

**Figure 2:**
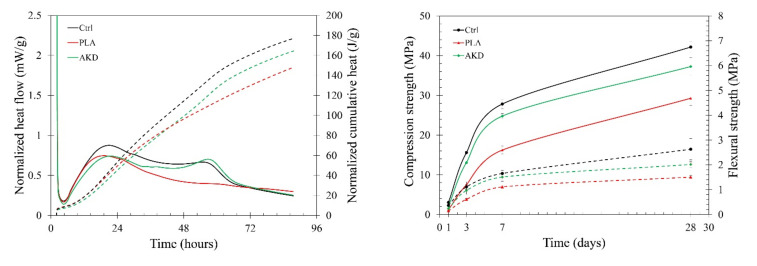
(**Left**) Isothermal calorimetry test results (solid lines represent the normalized heat flows; dotted lines represent the normalized cumulative heat curves); (**right**) strength test results at 1, 3, 7 and 28 days after casting (solid lines represent the compression strength; dotted lines represent the flexural strength).

**Figure 3:**
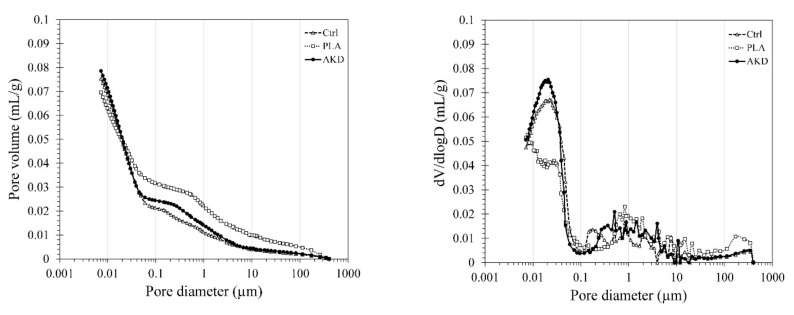
(**Left**) pore volume in relation to the pore size diameter distribution for the Ctrl, PLA and AKD specimens; (**right**) dV/dlogD in relation to the pore size diameter distribution for Ctrl, PLA and AKD specimens.

The results of the effect that both PLA and AKD healing agents had on the mortar functional properties (i.e., strength, hydration, workability, air void content and setting time) are reported in Figure 2 and Table 4, as well as the pore volume and pore size distribution of one specimen of each mixture (Figure 3).

The results of the isothermal calorimetry test show that the addition of both PLA and AKD lowers the heat development during the hydration of BFSC (Figure 2, left). During the first 10 h (induction or dormant phase), the heat released by the hydration of cement is comparable among the different mixtures. At around 24 h, the clinker peak of the Ctrl sample is higher than both those of PLA and AKD. After the first peak, a second (slag) peak is visible for the Ctrl hydration curve, as it is for AKD. On the contrary, no (relevant) slag peak is visible in the PLA hydration curve, which results in releasing the lowest amount of cumulative heat (e.g., 150 J/g) at around 84 h. The Ctrl mixture releases the highest amount of heat (e.g., reaching 180 J/g at around 84 h) overall, especially during the acceleration period (between 10 and 20 h). Lower heat is released by the AKD samples, as noticeable by the normalized cumulative heat curve. For AKD, the normalized cumulative curve suggests slight retardation of the hydration process, while the lack of the slag peak for the PLA hydration curve suggests a certain interaction between the healing agent and the matrix, compromising its hydration progress. The potential retardation effect that the addition of AKD healing agents has on the hydration of BFSC is further confirmed by the setting time results (Table 4). While the initial setting time of AKD and PLA is marginally different from that of the Ctrl (e.g., 185 ± 10 and 155 ± 15 min, respectively), the final setting time of AKD is 100 min higher (e.g., 510 ± 15 min). At the same time, the final setting of PLA occurs at 435 ± 10 min, slightly higher than the Ctrl (e.g., 410 ± 18 min). A certain influence upon the addition of both healing agents is also visible according to the results of the compression and flexural strength of each mortar mixture, as reported in Figure 2 (right). Visibly, Ctrl has the highest compression and flexural strength at any age of testing, reaching at 42.2 and 2.6 MPa, respectively, at 28 days. Slightly lower is the strength progress of AKD, of which the compression strength is between 15 and 30% lower than Ctrl at 1 and 3 days of age (e.g., 2.1 and 13.1 MPa, respectively) and around 10% lower at 7 and 28 days of age (e.g., 24.8 and 37.3 MPa, respectively). The addition of AKD in the BFS mixture also causes a reduction of flexural strength of around 10–30%, reaching a 28-day flexural strength equal to 2.0 MPa. The lowest compression and flexural strength are measured for the PLA mixture at any age of testing. The compression strength of PLA is equal to 40%, 48%, 58% and 69% of that of the Ctrl mixture at 1, 3, 7 and 28 days of age, respectively. Similarly, the flexural strength of PLA is equal to 40%, 55%, 67% and 57% of that of the Ctrl mixture at 1, 3, 7 and 28 days of age, respectively. Contrarily to the strength, setting time and isothermal calorimetry results, the effect of PLA and AKD healing agents on workability and air void content is negligible. The results of the flow table test (Table 4), conducted according to ASTM C143 [28], indicate that a slight increase of the mortar workability occurs since the flow of Ctrl, PLA and AKD is equal to 160 ± 10, 182 ± 14 and 177 ± 10 mm, respectively. A similar increase is noticeable on the air void content of the mixtures since, according to the results of the ASTM C185 test [29], the Ctrl, PLA and AKD have 4.1, 4.8 and 5.0% of air void, respectively.

The pore volume and the pore size distribution of Ctrl, PLA and AKD are reported in Figure 3. According to the MIP test, the three mixtures have comparable pore volume, equal to 0.0753, 0.0696 and 0.0786 mL/g for Ctrl, PLA and AKD, respectively. Both Ctrl and AKD mixtures are characterized by a significant number of small pores (e.g., 0.01–0.1 µm) and comparable distribution of the pore sizes. On the other hand, PLA has the highest number of larger pores (e.g., 0.1–100 µm), even though it has the lowest total pore volume.

### 3.2. Self-Healing Capacity

**Figure 4:**
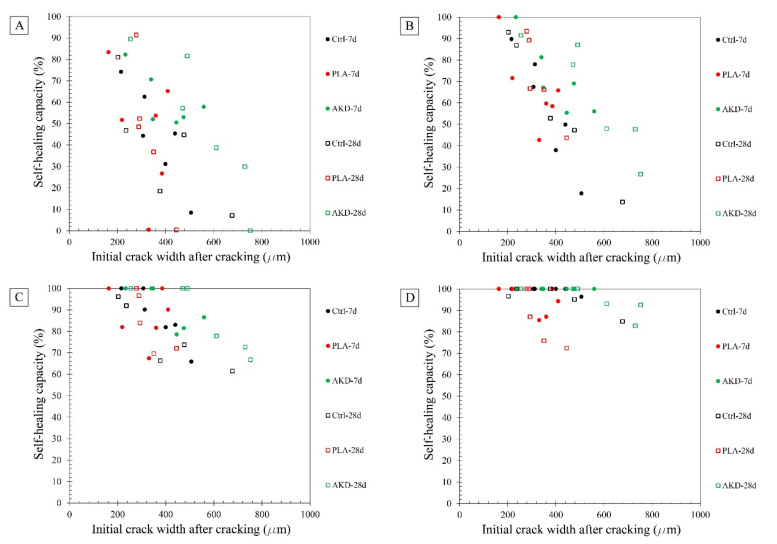
Self-healing capacity measured through optical microscopy analysis at 28 days (**A**), 56 days (**B**), 112 days (**C**) and 168 days (**D**) of self-healing. “Mixture-7d” refers to specimens cracked at 7 days of age, while “Mixture-28d” refers to specimens cracked at 28 days of age.

**Figure 5:**
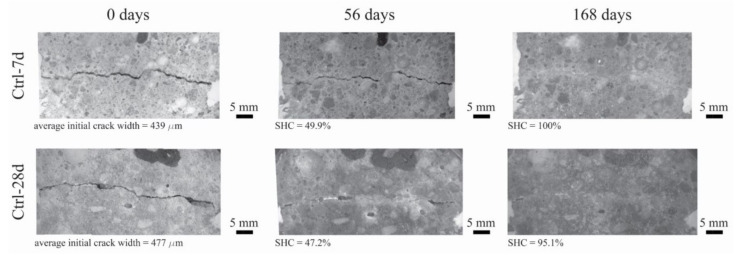
Optical microscope images (magnification 0.63×) of one specimen for Ctrl-7d (**top row**) and Ctrl-28d (**bottom row**) before and after 56 and 168 days of self-healing.

**Figure 6:**
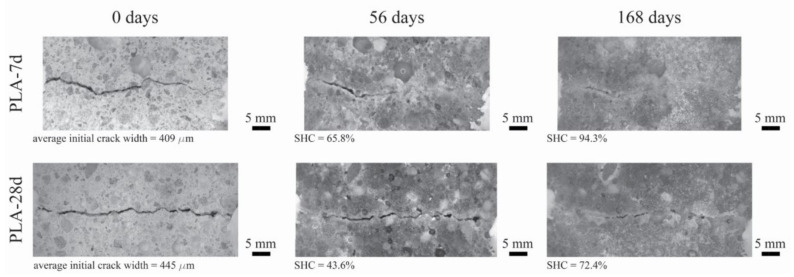
Optical microscope images (magnification 0.63×) of one specimen for PLA-7d (**top row**) and PLA-28d (**bottom row**) before and after 56 and 168 days of self-healing.

**Figure 7:**
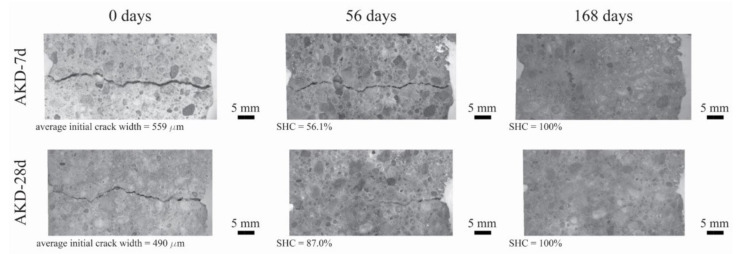
Optical microscope images (magnification 0.63×) of one specimen for AKD-7d (**top row**) and AKD-28d (**bottom row**) before and after 56 and 168 days of self-healing.

The results of the self-healing capacity through the optical microscopy analysis of each mortar mixture are reported in Figure 4, while the optical microscope images of the one selected specimen for each mixture (before and after 56 and 168 days of self-healing) are reported in Figure 5, Figure 6 and Figure 7.

Figure 4 clearly shows that the self-healing capacity of all of the specimens is directly proportional to the duration of the self-healing period. After 28 days of self-healing, no specimen has 100% SHC, regardless of its composition. Furthermore, a higher SHC is measured for smaller initial crack widths for all specimens. The 28 days’ SHC of the Ctrl and PLA mixtures is evenly distributed in the range of 200–400 µm, regardless the age of cracking of the specimens, while AKD mixtures have slightly better performance. At 56 days of self-healing, the SHC of all specimens overall uniformly improves. For crack widths ranging between 200 and 350 µm, most of the specimens have a SHC between around 70 and 95%, with two specimens (from PLA-7d and AKD-7d) reaching 100%. For crack widths ranging between 200 and 350 µm, no significant differences are visible between Ctrl and PLA specimens, of which the SHC overall ranges between 15 and 65%. At the same time AKD specimens have a SHC ranging between 25 and 85% for the same crack widths. At 112 days of self-healing, no specimen has a SHC lower than around 55%, regardless of the mixture composition, further confirming the proportional relation between self-healing capacity and incubation duration. At this time, the number of specimens with 100% SHC is equal to 2 for Ctrl-7d (maximum crack width of 307 µm), 0 for Ctrl-28d, 2 for PLA-7d (maximum crack width of 385 µm), 1 for PLA-28d (maximum crack width of 279 µm), 3 for AKD-7d (maximum crack width of 348 µm) and 3 for AKD-28d (maximum crack width of 490 µm). In the crack width range between 200 and 400 µm, the overall SHC of Ctrl and PLA specimens ranges between 65 and 95%, while all of the AKD specimens have 100% SHC. For crack widths between 400 and 800 µm, the SHC of all the AKD specimens (ranging between around 65 and 100%) is higher than that of the Ctrl specimens (ranging between around 60 and 80%). A total of 21 specimens have 100% SHC after 168 days of self-healing. At this time, the maximum crack width that is completely self-healed was 439 µm for Ctrl-7d, 378 µm for Ctrl-28d, 385 µm for PLA-7d, 289 µm for PLA-28d, 559 µm for AKD-7d and 490 µm for AKD-28d (as reported in Figure 4 and also visible in Figure 5, Figure 6 and Figure 7). Up to 500 µm crack width, all of the Ctrl specimens have 100% SHC apart from 3 specimens that have SHC higher than 90%. The AKD mixture is the best self-healing performing series, with all specimens with 100% SHC up to 559 µm wide cracks and with 3 specimens with SHC between 80 and 95% for cracks up to 800 µm wide. On the contrary, the PLA specimens have a more scattered behavior. Even though a SHC of 100% is measured for 3 PLA-7d specimens as well as for 2 PLA-28d specimens, 3 further specimens for each mixture have SHC of 70–95% for cracks ranging between around 300–420 µm.

**Figure 8:**
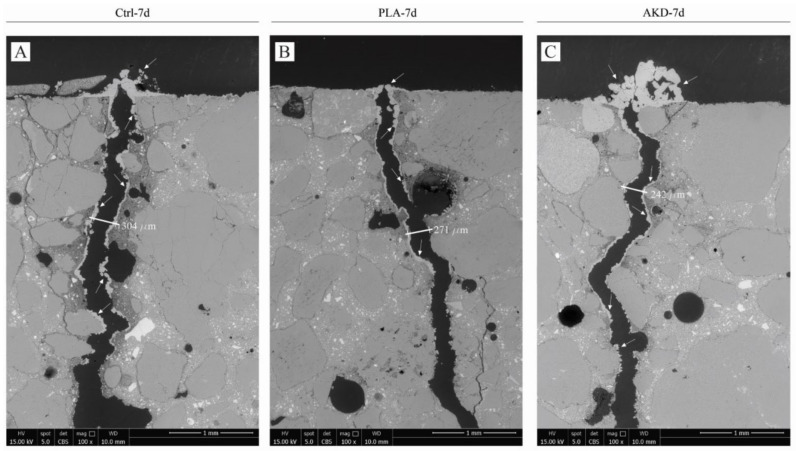
ESEM/BSE micrographs of the top portion perpendicular to the crack for specimens cracked at 7 days of age. (**A**) Ctrl-7d; (**B**) PLA-7d; (**C**) AKD-7d. White arrows indicate self-healing precipitates.

**Figure 9:**
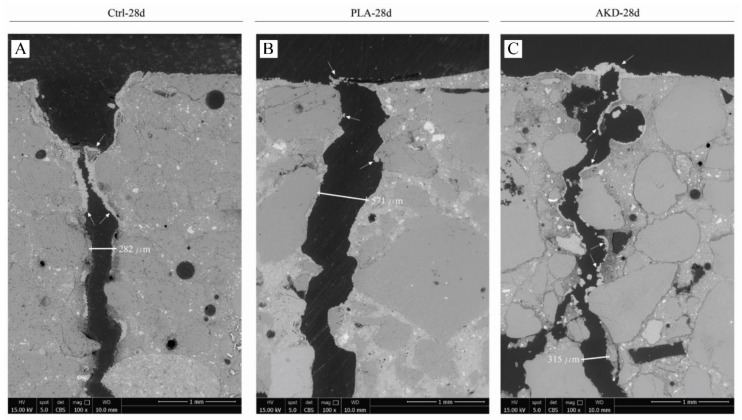
ESEM/BSE micrographs of the top portion perpendicular to the crack for specimens cracked at 28 days of age. (**A**) Ctrl-28d; (**B**) PLA-28d; (**C**) AKD-28d. White arrows indicate self-healing precipitates.

To observe the formation of self-healing precipitates in the crack depth, SEM analysis of the polished sections of one specimen for each mixture were also conducted, of which micrographs are reported in Figure 8 and Figure 9.

Further ESEM/BSE micrographs of self-healing precipitates’ formation along the crack depth are reported in Figure 8 and Figure 9, which confirm the superficial crack closure of the specimens. Complete crack closure occurs for Ctrl-7d, AKD-7d and AKD-28d, where a significant agglomeration of precipitates is observable at the crack mouth. For PLA-7d, Ctrl-28d and PLA-28d, the crack bridging layer of precipitates is visibly thinner or less compact, also presenting gaps that do not allow complete closure of the crack. For all the specimens, significant precipitation occurs mainly at the crack mouth, hence at the portion mostly exposed to the outside environment. Deeper in the crack, only thin layers of precipitates (e.g., around 20 µm thick) or localized agglomerates are observable at the crack sides for Ctrl-7d, PLA-7d, AKD-7d and AKD-28d, but not bridging the crack in its depth.

### 3.3. Freezing–Thawing Resistance of Carbonated Specimens

**Figure 10:**
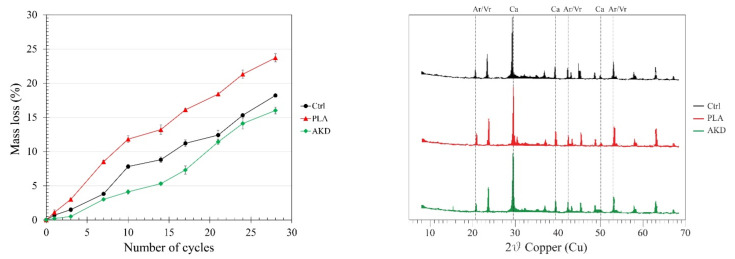
(**Left**) Mass loss due to 28 cycles (20 h at −20 °C and 4 h at 20 °C) of freezing–thawing of Ctrl, PLA and AKD specimens; (**right**) XRD spectrum of carbonated crystals identified from Ctrl (black), PLA (red) and AKD (green) powders. Ca and Ar/Vr indicate the representative peaks of calcite and aragonite/vaterite, respectively.

The carbonation depth results of specimens exposed to natural carbonation for 11 months as well as their mass loss due to freezing–thawing degradation and the XRD spectra of carbonated specimens are reported in Table 5 and Figure 10.

According to Figure 10, PLA is the most sensitive mixture to freezing–thawing attack. After the first 10 cycles, the mass loss of the PLA specimens is on average 11.8%, while that of Ctrl and AKD specimens is equal to 7.1 and 4.8%, respectively. At the end of the 28th freeze–thaw cycle, the total average mass loss of the PLA specimens is 23.6%, while for Ctrl and AKD it is equal to 18.2 and 16.0%, respectively. While PLA has the highest mass loss due to freezing–thawing after every cycle, the mass loss progress of Ctrl and AKD looks similar up to the 7th cycle and after the 21st. Between these two periods, a steeper mass loss is noticeable for the Ctrl specimens, while a higher mass loss is measured for AKD after the 21st cycle. No significant differences related to the carbonation depth are measured among the different mixtures, since CO_2_ on average penetrates up to 2.76, 2.87 and 2.61 mm in Ctrl, PLA and AKD specimens, respectively (Table 5). From the carbonated portions of all of the specimens, calcite and aragonite/vaterite crystals are identified, as confirmed by XRD analysis (Figure 10, right).

## 4. Discussion

### 4.1. Effect of Healing Agents on Mortar Functional Properties

The results reported in Section 3.1 show the influence that PLA and AKD healing agents have on some functional properties of BFSC paste and mortar, namely, the heat developed during hydration, compression and flexural strength, air void content, setting time, workability and porosity. As shown in Figure 2, the addition of PLA influences the cement hydration curve: the first peak of PLA is about 0.25 mW/g lower than that of the Ctrl mixture, while no second peak (related to the pozzolanic reaction of BFSC) is visible. In so doing, the cumulative heat at 86 h after mixing is about 40 J/g lower than that of the Ctrl mixture. As a consequence, both the compression and flexural strength of the PLA mortar are about 30–50% lower than that of the Ctrl mixture at any age of testing (e.g., 1, 3, 7 and 28 days). While PLA inclusion does not affect the mixture workability and setting time, it does influence the internal air system of the mixture. Compared to the Ctrl mixture, the air void content increases by 0.7% (Table 4) and the mortar microstructure gets coarser, as visible from the higher number of pores ranging between 0.05 and 100 µm (Figure 3). Similar results about the reduction of the slag reaction due to the inclusion of PLA healing agents were obtained by Mors and Jonkers [23]. As reported in their study, the influence that PLA inclusion has on BFSC hydration is caused by the relatively low alkalinity of the pore solution that leads to significant loss of strength and integrity when PLA is hydrolyzed [22]. Previous studies demonstrated that the inclusion of PLA in Ordinary Portland cement (OPC) mortars does not negatively affect the functional properties of the mixture, as observed in this study, hence suggesting the higher applicability of PLA healing agents in more alkaline mixtures [25,26].

The combination of the mortar alkaline environment and the inclusion of PLA or AKD healing agents increase the air void content from 4.0% (Ctrl) to 4.8% and 5.0% for PLA and AKD, respectively. Since some healing agent particles are unstable in highly alkaline environments, the interaction between biodegradable particles and the pore solution leads to the particles’ degradation via base catalyzed hydrolysis and the consequent formation of voids [32]. The time at which this mechanism can occur can vary from hours to weeks after casting, and the increase in the air void content of both PLA and AKD mixtures suggests that the degradation of (at least) a certain amount of healing agents already occurs during the early-age phase of the mixtures. Differently from PLA, AKD does only slightly affect the hydration of BFS cement by reducing the first peak by 0.25 mW/g, but preserves the second slag reaction peak, resulting in a cumulative heat equal to 165 J/g at 86 h after mixing (while that of the Ctrl is equal to 180 J/g). As a consequence, the strength development of AKD mortar is only about 10% lower than that of the Ctrl mixture at any testing age and the microstructure does not become coarsened. On the other hand, the results reported in Table 4 suggest that AKD serves as set retarding agent for BFS cement mortar, not affecting the initial setting time, but increasing the final one (about 100 min higher than that of the Ctrl mixture).

### 4.2. Effect of Healing Agents on Self-Healing Capacity

The results reported in Section 3.2 show the self-healing capacity of Ctrl, PLA and AKD mortars as a function of self-healing duration (e.g., 28, 56, 112 and 168 days), age of cracking (e.g., at 7 or 28 days after casting) and crack width (e.g., from 200 to 800 µm). In line with previous studies [10], Figure 4 clearly shows that the self-healing capacity of each specimen is proportional to the self-healing duration, and that smaller cracks require less time to become completely self-healed. Differently from other studies [9], no significant differences between the self-healing capacity of specimens cracked at 7 or 28 days after casting are visible. Overall, the AKD specimens have slightly higher self-healing capacity than both Ctrl and PLA specimens at each testing day, which developed similarly, especially until 56 days after casting. Regardless of the age of cracking, the maximum SHC was 81% for Ctrl specimens (crack width of 203 µm), 91% for the PLA specimens (crack width of 279 µm) and 89% for AKD specimens (crack width of 255 µm) after 28 days of self-healing. At 56 days, the maximum SHC was 93% for Ctrl specimens (crack width of 203 µm), 100% for the PLA specimens (crack width of 163 µm) and 100% for AKD specimens (crack width of 234 µm). The different performances of PLA and AKD specimens can be observed more in detail at 112 and 168 days after casting. After 112 days of self-healing, the largest width that specimens could self-heal was 307 µm for the Ctrl mixture, 385 µm for the PLA mixture and 490 µm for the AKD mixture. The self-healing capacity of PLA specimens remains lower than that of the Ctrl specimens, with 6 specimens out of 11 that did not reach 100% after 168 days of self-healing. At the same time, all of the AKD specimens with a crack width up to 559 µm self-healed completely, and those with a crack width between about 600 and 800 µm had a self-healing capacity between 80 and 95%. As a matter of fact, the maximum healable crack width of AKD specimens is 120 µm higher than that of the Ctrl specimens. These results suggest that PLA is less suitable to be applied as healing agent in BFSC mixtures than AKD, because of the highly alkaline environment that the former needs to be activated before becoming available for bacterial metabolic conversion. Differently, the bacterial conversion of alkanoates is not (that) dependent on the alkalinity of cementitious materials mixtures, and as such, the self-healing capacity of BFSC mortar increases upon the inclusion of AKD healing agents.

The better self-healing performance of AKD specimens is further demonstrated by the SEM micrographs reported in Figure 8 and Figure 9. A thick layer of self-healing precipitates is visible at the top of the AKD specimens cracked at both 7 and 28 days after casting. Thanks to the higher potential of continuous hydration of early-age cracked specimens, Ctrl-7d also had a sealing layer of crystals at the crack mouth, while the self-healing layers of Ctrl-28d and both PLA specimens could not completely bridge the crack sides. Nevertheless, the formation of self-healing crystals is limited to the top portion of the crack for all specimens. Besides the relatively thick layer of precipitates at the crack mouth for Ctrl-7d, PLA-7d, AKD-7d and AKD-28d, only 20–50 µm thick layers are visible inside the crack up to about 5 mm of depth. As already pointed out by others [10], the metabolic aerobic conversion of organic compounds depends on the availability of oxygen, which is obviously higher at the crack mouth rather than in the crack depth. In combination with higher availability of CO_2_ that triggers carbonation of the matrix at the same locations, the closure of the top portion of the crack further mitigates the ingress of oxygen deep in the crack, hence limiting more internal self-healing. In so doing, some functional properties that require complete filling of the whole crack (e.g., strength) cannot be recovered [33]. On the contrary, previous studies demonstrated that a thin layer of precipitates at the specimen surface can initially mitigate chloride penetration in the crack, hence partially restoring the chloride penetration resistance of uncracked specimens [26].

### 4.3. Freezing–Thawing Resistance of Carbonated Specimens

The results reported in Section 3.3 show the carbonation and freezing–thawing resistance of Ctrl, PLA and AKD mixtures. As visible from Table 5, the carbonation depth after 11 months of natural exposure was similar among the different specimens, with an average of 2.76 mm, 2.87 mm and 2.61 mm for Ctrl, PLA and AKD, respectively. The slightly higher carbonation depth measured for the PLA specimens is likely related to the relatively coarser microstructure of the mixture due to the healing agent inclusion, as demonstrated by the MIP test visible in Figure 3. The coarser microstructure of BFSC mortar with added-in PLA is also responsible for the lower resistance to freezing–thawing cycles. According to Figure 10, the mass loss due to the freezing–thawing of the PLA specimens is, on average, 5–10% higher than that of both Ctrl and AKD, with a mass loss after 28 cycles equal to about 24%. These results are in line with the findings of Mors and Jonkers [23], who observed that the inclusion of poly-lactic-based healing agents (e.g., PLA) increased the surface water absorption of BFSC concrete specimens. Hence, even though the air void content of the PLA mixture increased by 0.7% compared to that of the Ctrl mixture, the surface water uptake due to microstructural coarsening and consequent internal water freezing–thawing seems the main factor that reduces the resistance of the mixture. Differently, the inclusion of AKD healing agents did not coarsen the microstructure of the BFSC mortar (Figure 3), and indeed, the carbonation depth was, on average, 0.15 mm lower than that of the Ctrl mixture. Furthermore, the inclusion of AKD healing agents increased the air void content of the mixture by 0.9% compared to the Ctrl mixture. A higher air void content increases the resistance to freezing–thawing because it allows water to expand inside the matrix without stressing the surrounding mixture [34]. As a consequence, the overall mass loss due to the freezing–thawing of the AKD mixture was 2–5% lower than that of the Ctrl mixture, with a final mass loss after 28 cycles of 16% (i.e., 2% lower than that of the Ctrl).

It is worth mentioning that the air void content increase noticed upon the addition of AKD healing agents is likely the result of the degradation of some added-in particles, which, due to the alkaline environment of the matrix, leave empty voids, as previously discussed in Section 4.1. Nevertheless, the results reported in Section 3.2 demonstrated that the inclusion of AKD healing agents improves the self-healing capacity of BFSC mortar. Hence, even though some healing agent particles might be dissolved, the substrate needed for the metabolic activity of bacteria and consequent self-healing precipitation would still be available inside the matrix, enhancing the self-healing capacity of the mixture.

## 5. Conclusions

The aim of this study was to investigate the applicability of PLA and AKD healing agents in BFSC mortar mixtures by assessing their influence on mortar’s functional properties, self-healing capacity, resistance to carbonation, freezing–thawing and autogenous shrinkage. From the experimental campaign reported in the present study, the following major conclusions can be drawn:

- The inclusion of PLA influences the hydration of BFS cement, compromising the slag reaction of the paste. As a consequence, the compression and flexural strength drop. At 28 days, the compression and flexural strength values were 13 MPa and 1.4 MPa lower than those of the Ctrl mixture. On the other hand, the inclusion of AKD only slightly influenced the heat development during hydration of the mixture, with a consequent strength drop of around 10%. AKD significantly increased the final setting time of the mixture by about 100 min. Compared to the Ctrl mixture, the inclusion of PLA and AKD healing agents increased the air void content by 0.7% and 0.9%, respectively;
- The self-healing capacity of each specimen was proportional to the self-healing duration. Furthermore, fewer wide cracks were healed more easily and quickly. On the contrary, no significant differences were observed regarding the self-healing capacity of specimens cracked at 7 or 28 days after casting, regardless of their composition. The performance of Ctrl and PLA specimens was similar up to 112 days of self-healing, after which most of the Ctrl specimens self-healed completely, while 6 out of 11 PLA specimens did not. On the other hand, the AKD specimens had better self-healing performance than the others overall. The maximum completely healed crack width of the AKD specimens was 234 µm after 56 days, 490 µm after 112 days and 559 µm after 168 days. The latter was 114 µm and 159 µm higher than the maximum healed crack width for Ctrl and PLA specimens, respectively.
- SEM analysis showed that the top of the crack of Ctrl-7d, AKD-7d and AKD-28d were completely self-healed. Because of the lower concentration of CO_2_ and lack of oxygen, only 20–50 µm thick layer of precipitates were observed at the sides deeper in the crack, demonstrating that the most significant self-healing precipitation occurs at the specimens’ surface;
- The inclusion of PLA and AKD increased the air void content of BFSC mortar by 0.7% and 0.9%, respectively, compared to the Ctrl mixture. For the AKD mixture, this leads to higher resistance to freezing–thawing cycles in terms of mass loss. On the other hand, even though the air void content of PLA was higher than that of the Ctrl, the mass loss for PLA was 5.4% higher than that of the Ctrl mixture, likely because of the coarser microstructure caused by the inclusion of poly-lactic acid healing agents.

## Figures and Tables

**Table 1:** Composition of mixtures analyzed through isothermal calorimetry.

Mixture	Cement Type	Water/Cement	HA (% by Mass of Cement)
Ctrl	CEM III/B 42.5 N	0.5	0
PLA	CEM III/B 42.5 N	0.5	2.6
AKD	CEM III/B 42.5 N	0.5	2.6

**Table 2:** Mixing proportions of mortar specimens per kg of mixture.

Ingredients	Weight (g)
CEM III/B 42.5 N	220.9
Water	110.5
Aggregates (0.125–2 mm)	662.7
Healing agent	5.9 ^1^

^1^ only for bacteria-based mixtures.

**Table 3:** Fraction and particle size of aggregates per kg of mixture.

	2–1 mm	1–0.5 mm	0.5–0.25 mm	0.25–0.125 mm
Percentage (%)	33	34	21	12
Quantity (g)	218.7	225.3	139.2	79.5

**Table 4:** Results of workability, air void content and setting time tests of each mortar mixture. Each result is the average of three replicates.

	Workability (ASTM C143)	Air Void Content (ASTM C185)	Setting Time (EN 196-3)	
	Flow (mm)	Content (%)	Initial (min)	Final (min)
Ctrl	160 ± 10	4.1	165 ± 12	410 ± 18
PLA	182 ± 14	4.8	155 ± 15	435 ± 10
AKD	177 ± 10	5.0	185 ± 10	510 ± 15

**Table 5:** Average carbonation depth (mm) for Ctrl, PLA and AKD specimens.

	Ctrl	PLA	AKD
Average CO_2_ depth (mm)	2.76	2.87	2.61

## Data Availability

Not applicable.

## References

[1] Darquennes A., Roziere E., Khokhar M.I.A., Turcry P., Loukili A., Grondin F. (2012). Long-term deformations and cracking risk of concrete with high content of mineral additions. Mater. Struct..

[2] Luo R., Cai Y., Wang C., Huang X. (2003). Study of chloride binding and diffusion in GGBS concrete. Cem. Concr. Res..

[3] Dhir R.K., El-Mohr M.A.K., Dyer T.D. (1996). Chloride binding in GGBS concrete. Cem. Concr. Res..

[4] Itim A., Ezziane K., Kadri E.H. (2011). Compressive strength and shrinkage of mortar containing various amounts of mineral additions. Constr. Build. Mater..

[5] Dubovoy V.S., Gebler S.H., Klieger P., Whiting D.A., Frohnsdorff G. (1986). Effects of ground granulated blast-furnace slags on some properties of pastes, mortars and concretes. Blended Cements.

[6] Lanser P.A., Burger A.M. (2008). Carbon dioxide as a stimulus for life cycle thinking in cement and carbon neutral concrete building. Concr. Prod..

[7] Darquennes A., Staquet S., Espion B. (2011). Behaviour of slag cement concrete under restraint conditions. Eur. J. Environ. Civ. Eng..

[8] Briffaut M., Benboudjema F., D’aloia L., Bahrami B., Bonnet A. Analysis of cracking due to shrinkage restraint in a concrete tunnel. Proceedings of the Numerical Modeling Strategies for Sustainable Concrete Structures.

[9] Darquennes A., Olivier K., Benboudjema F., Gagné R. (2016). Self-healing at early-age, a way to improve the chloride resistance of blast-furnace slag cementitious materials. Constr. Build. Mater..

[10] De Belie N., Gruyaert E., Al-Tabbaa A., Antonaci P., Baera C., Bajare D., Jonkers H.M. (2018). A review of self-healing concrete for damage management of structures. Adv. Mater. Interfaces.

[11] Hearn N. (1998). Self-sealing, autogenous healing and continued hydration: What is the difference?. Mater. Struct..

[12] Yoon I.S., Schlangen E. (2014). Experimental examination on chloride penetration through micro-crack in concrete. KSCE J. Civ. Eng..

[13] Ismail M., Toumi A., François R., Gagné R. (2008). Effect of crack opening on the local diffusion of chloride in cracked mortar samples. Cem. Concr. Res..

[14] Sahmaran M. (2007). Effect of flexure induced transverse crack and self-healing on chloride diffusivity of reinforced mortar. J. Mater. Sci..

[15] Wang J., Soens H., Verstraete W., De Belie N. (2014). Self-healing concrete by use of microencapsulated bacterial spores. Cem. Concr. Res..

[16] Wang J.Y., Snoeck D., Van Vlierberghe S., Verstraete W., De Belie N. (2014). Application of hydrogel encapsulated carbonate precipitating bacteria for approaching a realistic self-healing in concrete. Constr. Build. Mater..

[17] Wang J., Mignon A., Trenson G., Van Vlierberghe S., Boon N., De Belie N. (2018). A chitosan based pH-responsive hydrogel for encapsulation of bacteria for self-sealing concrete. Cem. Concr. Compos..

[18] Xu H., Lian J., Gao M., Fu D., Yan Y. (2019). Self-healing concrete using rubber particles to immobilize bacterial spores. Materials.

[19] Palin D. (2017). A Cost-Effective Bacteria-Based Self-Healing Cementitious Composite for Low-Temperature Marine Applications. Ph.D. Thesis.

[20] Palin D., Wiktor V., Jonkers H. (2015). Autogenous healing of marine exposed concrete: Characterization and quantification through visual crack closure. Cem. Concr. Res..

[21] Palin D., Wiktor V., Jonkers H. (2017). A bacteria-based self-healing cementitious composite for application in low temperature marine environments. Biomimetics.

[22] Mors R.M., Jonkers H.M. (2017). Feasibility of lactate derivative based agent as additive for concrete for regain of crack water tightness by bacterial metabolism. Ind. Crops Prod..

[23] Mors R. (2017). and Jonkers, H. Effect on concrete surface water absorption upon addition of lactate derived agent. Coatings.

[24] Vermeer C.M., Rossi E., Tamis J., Jonkers H.M., Kleerebezem R. (2021). From waste to self-healing concrete: A proof-of-concept of a new application for polyhydroxyalkanoate. Resour. Conserv. Recycl..

[25] Rossi E., Vermeer C.M., Mors R.M., Kleerebezem R., Copuroglu O., Jonkers H.M. (2021). On the applicability of a precursor derived from organic waste streams for bacteria-based self-healing concrete. Front. Built Environ..

[26] Rossi E., Roy R., Copuroglu O., Jonkers H.M. (2022). Influence of self-healing induced by polylactic-acid and alkanoates-derivates precursors on transport properties and chloride penetration resistance of sound and cracked mortar specimens. Constr. Build..

[27] (2009). Methods of Testing Cement—Part 1: Determination of Strength.

[28] (2020). Standard Test Method for Slump of Hydraulic-Cement Concrete.

[29] (2020). Standard Test Method for Air Content of Hydraulic Cement Mortar.

[30] (2009). Methods of Testing Cement—Part 3: Determination of Setting Times and Soundness.

[31] Rodríguez C.R., de Mendonça Filho F.F., Mercuri L., Gan Y., Rossi E., Anglani G., Antonaci P., Schlangen E., Šavija B. (2020). Chemo-physico-mechanical properties of the interface zone between bacterial PLA self-healing capsules and cement paste. Cem. Concr. Res..

[32] Niaounakis M. (2015). Biopolymers: Applications and Trends.

[33] Ferrara L., Van Mullem T., Alonso M.C., Antonaci P., Borg R.P., Cuenca E., De Belie N. (2018). Experimental characterization of the self-healing capacity of cement based materials and its effects on the material performance: A state of the art report by COST Action SARCOS WG2. Constr. Build. Mater..

[34] Winter N.B. (2012). Understanding Cement: An Introduction to Cement Production, Cement Hydration and Deleterious Processes in Concrete.

